# Overview of Structural and Functional Insights of SLFN12 Associated With Different Diseases

**DOI:** 10.7759/cureus.59515

**Published:** 2024-05-02

**Authors:** Mayasim Tilmisani, Safiah Alhazmi, Hind ALnajashi, Reem Alyoubi

**Affiliations:** 1 Department of Biological Sciences, King Abdulaziz University, Jeddah, SAU; 2 Blood Transfusion Services, King Abdulaziz University Hospital, Jeddah, SAU; 3 Central Lab of Biological Sciences, Faculty of Sciences, King Abdulaziz University, Jeddah, SAU; 4 Immunology Unit, King Fahad Medical Research Centre, King Abdulaziz University, Jeddah, SAU; 5 Neurology Division, Department of Internal Medicine, Faculty of Medicine, King Abdulaziz University, Jeddah, SAU; 6 Pediatric Neurology Division, Department of Pediatrics, Faculty of Medicine, King Abdulaziz University, Jeddah, SAU

**Keywords:** schlafen gene family, autoimmune diseases, multiple sclerosis, slfn12, schlafen12

## Abstract

Schlafen12 is a member of the Schlafen gene family where *Slfns* have been linked to many functions such as anti-proliferation and cell differentiation, viral replication inhibition, migration of cancer cells and invasion prevention, and sensitivity to DNA-damaging medicines. Researchers are interested in studying the biochemical mechanisms that control thymocyte development to extract and describe gene expression and transcriptionally elevated by the process of positive selection that led to the discovery of this novel gene family. This review aims to give adequate knowledge about human *SLFN12* by reviewing the most notable papers from five reliable databases regarding *SLFN12* milestones and alterations in *SLFN12 *expression in various disease discoveries from 1997 to the present. In conclusion, *SLFN12* seems to be linked with autoimmune diseases such as multiple sclerosis. Furthermore,* SLFN12* levels could modify the effects of radiation and chemotherapy.

## Introduction and background

Introduction

**Figure 1:**
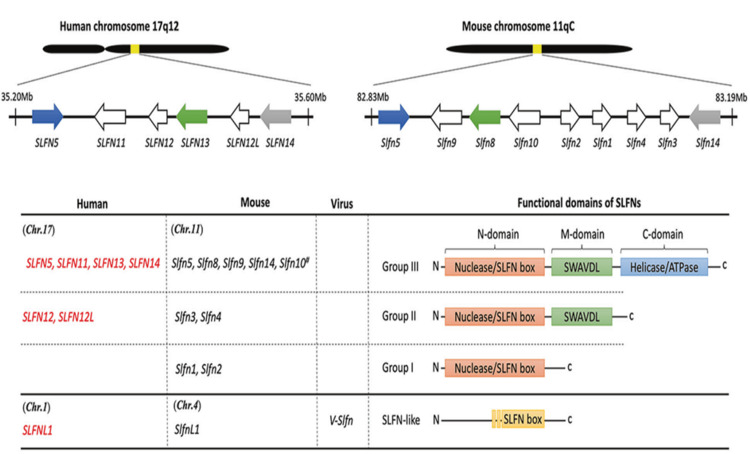
The SLFN genes of humans, mice, and viruses are compared according to their location and functional domains. Source: [2].

Over the last two decades, most research focused on the Schlafen (*SLFN*) family of proteins has concentrated on humans and mice. These proteins belong to clusters of genes that have been shown to be evolutionarily conserved across a broad variety of vertebrate species and are thought to have originated from a common ancestor through several unequal recombination processes [1]. Human *SLFN* genes (*SLFN5, SLFN11, SLFN12, SLFN12L, SLFN13*, and *SLFN14*) are found on chromosome 17, while Slfn genes (*Slfn1, Slfn2, Slfn3, Slfn4, Slfn5, Slfn8, Slfn9*, and *Slfn14*), as well as the pseudogene (*Slfn10*) are found on chromosome 11 in mice (Figure 1) [2]. Additionally, it has been shown that other species, such as orthopoxvirus, mice, and humans, possess a gene similar to *SLFN* (*SLFN*-like gene) that has a partially conserved SLFN box within their genomes. Moreover, proteins from the Schlafen family (*Slfns*) are found in many vertebrate species, including mammals, fish, and amphibians. Twenty years ago, *Slfn1*, 2, 3, and 4 were discovered to be differently controlled throughout T cell development and thymocyte maturation [3]. The German word Schlafen means "to sleep" and is derived from the Slfn1 behavior that pauses the cell cycle during G0/G1. Ever since, *Slfns* have been linked to functions such as anti-proliferation and cell differentiation, viral replication inhibition, migration of cancer cells and invasion prevention, and sensitivity to DNA-damaging medicines [3]. The categorization of SLFN proteins is based on the structural and functional characteristics of their domains, resulting in the identification of three distinct categories. The first cluster has a shared N-domain region with a nuclease structure and a distinctive *SLFN* box that is conserved across all SLFN proteins. The second group has an N-domain and a linker middle domain region, referred to as the M-domain, which harbors a SWAVDL motif that exhibits a putative protein-interacting region 14. The third group of SLFNs has the highest level of numerical representation among the various subgroups. The third functional putative helicase/ATPase C-terminal domain is composed of Walker A/B motifs. The biological role of SLFN-like proteins remains ambiguous, despite the presence of conserved amino acid sequences containing the SLFN box [1]. The Schlafen gene family has 10 members in mice and six members in humans. Schlafen proteins provide a variety of metabolic roles, ranging from translation inhibition (*SLFN12*) through tRNA cleavage prevention (*Slfn2*) [4]. The great majority of family members play physiological and metabolic roles that are mostly unclear. To our knowledge, no previous studies have focused on human *SLFN12*. As a result, this review aims to give adequate knowledge about human *SLFN12* by reviewing the most notable papers regarding *SLFN12* milestones and alterations in *SLFN12* expression in various disease discoveries from 1997 to the present.

Study design

Information is gathered from internet sources such as NCBI, PubMed, GeneCards, OMIM, and Google Scholar. The search is limited to English-language articles published between 1997 and 2023, and it is about 22 articles. The search is based on keywords that include gene and protein scientific names and symbols. The scientific full name is Schlafen Family Member 12, whereas the scientific symbol is *SLFN12*. It is also known as ribonuclease SLFN12, FLJ10260, EC 3.1, and *SLFN3*.

## Review

Mouse and human *SLFN12*

SLFN12 Milestone Discoveries

*SLFN12* belongs to the Schlafen gene family, which is found on chromosome 11 of the mouse genome. The *SLFN* gene family was discovered as a crucial component for thymocyte development in mice by Schwarz et al. in 1998 [5]. Researchers aimed to identify and define genes differently expressed and transcribed upregulated due to the positive selection method used to better understand the biochemical processes that drive thymocyte formation. By doing so, they discovered a novel gene family whose members are differently controlled throughout thymocyte formation [6]. Subtractive hybridization was done with thymus cDNA libraries generated from AND.4R and AND.B6 mice to discover genes that are increased due to selection that is positive. The discovery presented in this study demonstrates the presence of a previously unidentified gene that is regulated by developmental processes and has a higher level of expression in AND.B6 thymocytes compared to AND.4R thymocytes [7]. The nucleotide and amino acid sequences derived from the gene known as Schlafen1 (*Slfn1*) were utilized in a search using the gapped-basic local alignment search resource (BLAST) to explore the expressed sequence tag (EST) information databases [8]. As a result of employing this methodology, an additional three *Slfn* genes were isolated, exhibiting either likeness with 53 EST entries. The allocation of these ESTs to six separate genes has been determined using a sequence comparison. In addition, a segment of the *Slfn3* complementary DNA (cDNA) was employed for the purpose of screening a genomic library of mice. This screening process resulted in the identification of many clones that encode either the entire *Slfn3* gene or a partial sequence of it, as well as clones corresponding to *Slfn4, Slfn6*, and an extra clone labeled as *Slfn7*. Based on the aforementioned data, it is postulated by researchers that the Slfn family encompasses a minimum of seven distinct genes [7]. Subsequently, reverse transcription-polymerase chain reaction (RT-PCR) was employed to validate the expression of the recently discovered *slfn* genes. This was accomplished by utilizing cDNA derived from interferon (IFN)-stimulated bone marrow-derived macrophages obtained from B6 and 129 mice. The resulting amplified DNA fragments were then cloned in their entirety. The findings indicate that the genes *slfn5, slfn8, slfn9*, and *slfn10* possess four coding exons that exhibit identical structural characteristics. Notably, the last two exons of these genes encode protein components that are exclusive to the third subgroup of the Slfn protein family [9]. Slfns are commonly categorized into three distinct subgroups, which are determined by their size and composition. The size of Subgroup I Slfn isoforms ranges from 37 to 42 kDa, while Subgroup II Slfns have a size range of 58 to 68 kDa. The biggest members of the Slfn family (100-104 kDa) are found in Subgroup III, and they exhibit motifs that are similar to those present in the helicase superfamily I and the UvrD DNA helicase superfamily [10]. *Slfns* in mice are present in all three subgroups, but SLFNs in humans are found in subgroups II (*SLFN12, SLFN12L*) and III (*SLFN5, SLFN11, SLFN13, SLFN14*). The only orthologs shared by mice and men are *Slfn5/SLFN5* and *Slfn14/SLFN14*, which is consistent with the gene family's rapid evolution, particularly in mice [11].

SLFN12 Gene Expression

**Figure 2:**
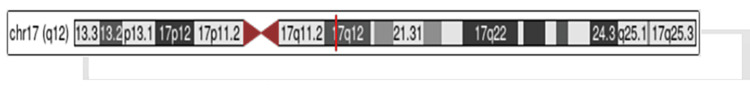
SLFN12 cytogenetic location in humans. *SLFN12* cytogenetic location in the human genome (17q12), in which the *SLFN12* gene is located on the long arm (q) of chromosome 17 at position 1, band 2.

The SLFN12 gene in humans is found on chromosome 17, specifically on 17q12 (Figure 2). Schwarz et al. (1998) used subtractive hybridization with mouse thymus cDNA clone encoding *Slfn1 *to discover upregulated mouse genes, *Slfn1, Slfn2, Slfn3*, and *Slfn4*, followed by database analysis. Northern blot analysis indicated that all four genes had low expression. *Slfn1, Slfn2*, and *Slfn4* were shown to be most abundant in the thymus, lymph node, and spleen, according to an RT-PCR study. Moreover, human *SLFN12* and *SLFN12L* have been shown to be orthologous to mouse *Slfn1, Slfn2, Slfn3*, and *Slfn4* [12].

SLFN12 Protein Structure

**Figure 3:**
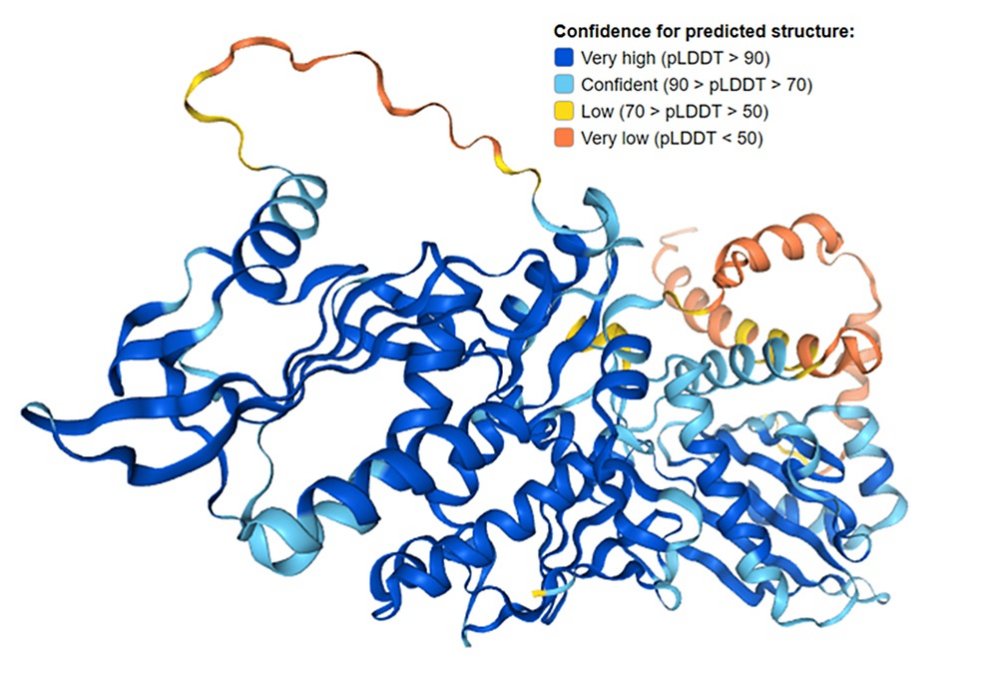
Structure prediction of Q8IYM2 (ribonuclease SLFN12 consists of 578 amino acids) Source: Alphafold project, version 2. Protein structure - SLFN12 - The Human Protein Atlas [15].

The Schlafen SLFN12 proteins exhibit a diverse array of metabolic functions, encompassing the inhibition of translation as well as the prevention of tRNA breakage induced by reactive oxygen species (ROS). Nevertheless, there is still a lack of comprehensive understanding of the physiological and metabolic roles of the members of the family [13]. In addition, it is worth noting that the *SLFN* family includes a total of nine genes in mice and six genes in humans. These genes possess an AAA ATPase-like domain at their N-termini and an adjacent domain known as "SWADL" (Ser-Trp-Ala-Asp-Leu), which has a high degree of conservation (Figure 3). The cellular location of SLFN proteins exhibits variations, suggesting their potential significance in biological processes. The cytoplasmic *Slfns *in mice are mostly populated by Group 1 and II *Slfns*, whereas Group III *Slfns* are largely localized within the nucleus. In comparison to the *Slfn* genes found in mice, the *SLFN* genes in humans only code for polypeptides belonging to Group II (*SLFN12*) and Group III (*SLFN5, SLFN11, SLFN13*, and *SLFN14*). Furthermore, it should be noted that *SLFN12* and *SLFN13* exhibit cytoplasmic localization, whereas *SLFN11, SLFN14*, and SLFN5 possess nuclear localization signals [1]. Immunofluorescence and immunostaining techniques predominantly localize *SLFN11* within the nucleus. However, it is noted that *SLFN11* also exerts influence on the control of proteotoxic stress and protein translation within the cytoplasmic area [14].

SLFN12 Functions

*SLFN12* orthologs in the mouse. Schwarz et al. (1998) discovered that mouse *Slfn1 *decreases cell proliferation and thymic development by interfering with thymocyte division. It was concluded that the SLFN protein family has a role in both cell proliferation and T-cell development [7]. Moreover, mitogen-driven induction of cyclin D1 (CCND1) was discovered as a target of mouse *Slfn1 *in its potential to produce a cell cycle arrest in the mid-G1 phase [6]. *Slfn4* mRNA levels in mice were shown to be upregulated during macrophage activation but downregulated during differentiation. In vivo, constitutive *Slfn4* expression in the myeloid lineage disrupted myelopoiesis. *Slfn4* downregulation during macrophage differentiation was hypothesized to be required for the formation of this lineage [12].

*SLFNs* have a role in immune cell development. The regulation of human *SLFN* members exhibits variability during the differentiation process from monocytes to dendritic cells (DCs). The downregulation of *SLFN12* implies that it may possess a deleterious role in the development of DCs. In contrast, the expression of *SLFN12* is increased throughout the process of T-cell activation [16].

*SLFNs* control viral replication and pathogenicity. Interferon-stimulated genes (ISGs) refer to a class of genes that are induced by IFNs and play a significant part in the process of viral propagation. Due to the observed induction of SLFN expression by type I IFN, SLFNs are categorized as ISGs possessing antiviral properties. The SLFN proteins have been observed to commonly target HIV (human immunodeficiency virus) and camelpox viruses [17,18].

*SLFNs* improve cancer cell drug sensitivity. Topoisomerase (TOP) inhibitors, a kind of DNA-damaging agent, play a significant role in oncology. Previous research has found that the level of *SLFN* expression corresponds with cancer cell responses to TOPs. SLFN11 is a crucial gene in the SLFN family that makes cancers more sensitive to chemotherapeutics. Cells exhibiting elevated levels of SLFN11 expression exhibit a notable reduction in proliferation subsequent to irinotecan treatment in comparison to cells with lower *SLFN11* expression levels [19]. *SLFN12*, like *SLFN11*, increases the medication sensitivity of malignant tumors. When coupled with phosphodiesterase 3A (*PDE3A*), *SLFN12* can increase tumor sensitivity to 6-(4-(diethylamino)-3-nitrophenyl)-5-methyl-4,5-dihydropyridazin-3(2H)-one (DNMDP) in lung adenocarcinoma cell lines [13].

*SLFN12* and sensitivity to DNA-damaging medicines. In triple-negative breast cancer (TNBC), *SLFN12* expression correlates with survival. However, *SLFN12* improves TNBC susceptibility to DNA-damaging drugs by lowering CHK1/2 phosphorylation, at least in part. This may enhance the survival of those with TNBC that overexpresses *SLFN12*. As a result, *SLFN12* levels may be utilized to modify or anticipate the effects of radiation and chemotherapy in TNBC [20].

SLFN12-Linked Diseases

*SLFN12* and allergic rhinitis symptoms. Researchers discovered a novel pattern for DNA methylation in *SLFN12*. While the direction of DNA methylation change in response to allergen exposure was associated with symptoms, they found no link between baseline DNA methylation and the onset of symptoms. Pyrosequencing and qPCR demonstrated connections between *SLFN12* DNA methylation, gene expression, and allergy symptoms, as well as variations in the baseline that predicted symptom severity [21].

*SLFN12* and DNA methylation in multiple sclerosis (MS). The downregulation of *SLFN12* has been seen in primary human cells subsequent to T-cell activation. The CD4 and CD8 subsets of T cells are important in the pathophysiology of MS. Hypermethylation of DNA, particularly the *SLFN12* gene, was observed with the use of type I IFNs, which is one of the approved treatment options for MS. Patients who had never been treated or off-treat for a prolonged period expressed downregulation of the *SLFN12* gene. Given these findings, the gene *SLFN12* emerges as a physiologically feasible candidate of interest for further exploration in the context of MS [22].

HIV-1 reactivation is inhibited by *SLFN12. *The Schlafen 12 protein (SLFN12) has been identified by researchers as a significant HIV-1 restriction factor. It exerts its effect by inducing a post-transcriptional barrier in cells infected with HIV-1, leading to a decrease in viral replication and a reduction in the reactivation of the virus from latently infected cells. The researchers employ an RNA FISH-flow experiment to establish that the upregulation of *SLFN12* is observed in HIV-1-infected cells that exhibit the presence of HIV-1 transcripts but lack the production of HIV-1 proteins. Therefore, the suppression of HIV-1 protein translation resulting from codon use plays a role in latency and can impede viral release following the reactivation of latent infection [23].

*PDE3A-SLFN12* complex and tumor cells. Molecular glues refer to a class of tiny molecular drugs that facilitate protein-protein interactions, resulting in either the degradation or stabilization of the target protein. A collection of chemical substances, including 17-β-estradiol (E2), anagrelide, nauclefine, and DNMDP, have been observed to induce apoptosis through the formation of complexes involving PDE3A and SLFN12. This is accomplished by the adherence to the enzymatic pocket of PDE3A, which enables the binding of compound-bound PDE3A and subsequent stabilization of SLFN12. Consequently, protein translation is inhibited, leading to the induction of apoptosis [13].

Limitations of this study

There are a few limitations to the review that should be considered: many studies have shown alterations in SLFN12 gene expression in various diseases. However, there was a shortage of information on SLFN12-related molecular pathways. Furthermore, we cannot totally rule out the influence of the differentiation process in immune cells or the response to medications on variations in SLFN12 gene expression in diseases. Despite these limitations, this study has offered a broad background as well as primary findings concerning SLFN12-linked diseases, which may lead to more research.

## Conclusions

This review sought to better understand the 12th member of the *SLFN* family by evaluating the existing data on *SLFN12*. Based on the prior literature, this article's review suggested that *SLFN12* might have a function in immune cell differentiation, apoptosis, control viral replication and pathogenicity, increase tumor sensitivity to chemotherapeutics, and *SLFN12* levels may be utilized to modify or anticipate the effects of radiation and chemotherapy. Furthermore, *SLFN12* seems to be linked with autoimmune diseases such as MS. However, a specific process through which *SLFN12* may contribute to the development of autoimmune diseases remains in question and needs further research.
